# A report on the use of a single intra-articular administration of autologous platelet therapy in a naturally occurring canine osteoarthritis model - a preliminary study

**DOI:** 10.1186/s12891-020-3140-9

**Published:** 2020-02-27

**Authors:** J. C. Alves, A. Santos, P. Jorge, C. Lavrador, L. Miguel Carreira

**Affiliations:** 1 Divisão de Medicina Veterinária, Guarda Nacional Republicana (GNR), Rua Presidente Arriaga, 9, 1200-771 Lisbon, Portugal; 2 MED – Mediterranean Institute for Agriculture, Environment and Development, Instituto de Investigação e Formação Avançada, Universidade de Évora, Pólo da Mitra, Ap. 94, 7006-554 Évora, Portugal; 3 Faculty of Veterinary Medicine, University of Lisbon (FMV/ULisboa), Lisbon, Portugal; 4 Interdisciplinary Centre for Research in Animal Health (CIISA), University of Lisbon (FMV/ULisboa), Lisbon, Portugal; 5 Anjos of Assis Veterinary Medicine Centre (CMVAA), Barreiro, Portugal

**Keywords:** Animal model, Dog, Osteoarthritis, Pain, Autologous platelet concentrate, Clinical metrology instruments

## Abstract

**Background:**

Osteoarthritis (OA) represents a significant burden to societies, as it affects quality of life, performance and poses a large healthcare cost. We aimed to describe the use of a single intra-articular (IA) injection of an autologous platelet therapy in the management of osteoarthritis (OA) in a naturally occurring canine model.

**Methods:**

Fifteen police working dogs with bilateral hip OA were treated with 3 ml of platelet concentrate per hip joint, produced with the V-PET kit. Response to treatment was measured by the Canine Brief Pain Inventory (CBPI, divided in pain interference score – PIS, and Pain Severity Score - PSS), Liverpool Osteoarthritis in Dogs (LOAD), Canine Orthopedic Index (COI, divided in four dimensions: function, gait, stiffness and quality of life - QOL) and the Hudson Visual Analogue Scale (HVAS). Seven different time points were considered: T0 (before treatment), T1 (after 15 days), T2, T3, T4, T5 and T6 (after 1, 2, 3, 4 and 5 months respectively). Results from each evaluation moment were compared with T0 with a Paired Samples T-Test, and a *p* < 0.05 was set.

**Results:**

Significant differences were observed at T1 (*p* < 0.01 for HVAS, PSS, COI, Gait and QOL; *p* = 0.01 for PIS; *p* = 0.02 for Function; and *p* < 0.05 for Stiffness), T2 (*p* < 0.01 for PSS, PIS and Gait; *p* = 0.01 for COI; *p* = 0.02 for HVAS, Function and QOL; and *p* = 0.04 for Stiffness), T3 (*p* < 0.01 for HVAS, PSS, PIS, Function and Gait; *p* = 0.01 for COI; and *p* = 0.02 for QOL), T4 (*p* < 0.01 for PSS; *p* = 0.03 for PIS and Gait), T5 (*p* < 0.01 for COI, Function and Gait; *p* = 0.03 for PSS, PIS and Stiffness), T6 (*p* < 0.01 for PSS, Function and Gait; *p* = 0.04 for PIS; *p* < 0.05 for COI) and T7 (*p* < 0.01 for PSS, Function and Gait; *p* = 0.01 for COI; and *p* < 0.05 for PIS).

**Conclusions:**

Autologous platelet therapy was used without apparent harm in the subjects. A single administration produced significant improvements, which lasted several months, and therefore warrants further study.

## Background

Osteoarthritis (OA) represents a significant burden to societies, as it affects quality of life, performance and poses a large healthcare cost [1]. It is also the most prevalent musculoskeletal disease in dogs, with an expected increase, due to a simultaneous increase in life expectancy and obesity [2]. For these reasons, it raises major welfare challenges and concern [3]. Translational research is a critical step towards understanding the long-term effects of OA, and animal models provide relevant ways to study the natural history and response to treatment [4]. Canine OA models have the advantages of being anatomically, biochemically, genomically and molecularly similar to humans, with close clinical progression and response to treatment. These naturally occurring models may better reflect the complex genetic, environmental, temporal and physiological influences present in humans, being the closest to a gold standard [4–9]. Therefore, exploring spontaneous OA in dogs under the One Medicine initiative can help improve the health and well-being of both humans and dogs [9, 10].

Pain and functional ability are important parameters in the evaluation of OA treatment efficacy [11]. The gold standard for the evaluation of lameness is through gait analysis [12] but this equipment is often confined to research facilities [9]. Several clinical metrology instruments (CMI) have been developed in order to measure outcome assessments, which for dogs are normally completed by a proxy. In human medicine, they are a standard, validated and accepted method for measuring chronic pain, and have formed an important part of the patient clinical assessment for over 30 years [13, 14]. The best ones developed for dogs, and that have been reported to have criterion validity, are the Canine Brief Pain Inventory (CBPI) and the Liverpool Osteoarthritis in Dogs (LOAD) [9, 14–16]. The CBPI is divided in two sections, a pain severity score (PSS), that assesses the magnitude of the animal pain, and a pain interference score (PIS), that assesses the degree in which pain affects daily activities [17]. The Canine Orthopaedic Index (COI) was developed to assess four domains in dogs with OA: stiffness, gait, function and quality of life (QOL). It has been shown to have excellent reliability and validity, and has been used to evaluate working dogs [18, 19]. Visual Analogue Scales are one of the techniques used to score pain and assess its severity, allowing the comparison of analgesic protocols. The Hudson Visual Analogue Scale (HVAS) has been deemed as repeatable and valid to assess the degree of mild to moderate lameness in dogs, compared with force plate analysis as a criterion-referenced standard [20].

Autologous platelets are a regenerative treatment modality for OA, used with the aim to stimulate the natural healing cascade and regeneration of tissues by a supraphysiologic release of platelet derived factors directly at the treatment site, without the risk of immune rejection or disease transmission [21–23]. Growth factors affect nearly every biological process [24] and, in platelets concentrates, insulin-like growth factor (IGF-1), transforming growth factor-β (TGF-β), platelet-derived growth factor (PDGF), vascular endothelial growth factor (VEGF) and basic fibroblast growth factor (b-FGF), signal cells to proliferate and influence their maturation, differentiation and tissue repair [25, 26]. Growth factors can be obtained from other sources, such as autologous conditioned plasma, and are able to reduce pain and lameness scores, and increase weight bearing when injected into OA joints [27–29]. In dogs, a single intra-articular (IA) PRP injection has resulted in clinical improvements for 12 weeks, in some cases without progression of radiographic signs [27, 30, 31]. Through this period, radiographic scores were the same as assigned before treatment [30]. Multiple injections protocols have also been described, providing improvements in ROM, pain, lameness and kinetics. Authors associated this response to treatment to an anti-inflammatory activity of PRP rather than any effect on tissue anabolism or catabolism [32]. It has also been used as a part of surgical protocols, leading to a significant improvement in gait performance in the postoperative period [31, 33]. V-PET is a platelet concentrate as well as conditioned plasma, which contain many autologous anti-inflammatory mediators and growth factors, reported to reduce pain and lameness scores and increase weight bearing in dogs with OA [27, 28].

The objective of this report is to describe the use of the platelet concentrate V-PET in the management of OA in a naturally occurring canine model. We hypothesize that a single IA administration of platelet concentrate can reduce pain scores in police working dogs with naturally occurring hip OA for a long period of time.

## Methods

The sample comprised animals selected from the population of police working dogs of the Guarda Nacional Republicana (Portuguese Gendarmerie Canine Unit). Selection was made by the assisting veterinarian, based on the dog’s history, trainer complaints, physical and radiographic findings consistent with bilateral naturally occurring mild and moderate hip OA, classified according to the Orthopedic Foundation for Animals scoring. Animals with other illnesses or under any other treatment were not included in the study, and were ruled out through physical examination, complete blood count and serum chemistry profile. Written, informed consent was obtained for all animals.

The animals were placed under light sedation using medetomidine (0.01 mg/kg) and buthorphanol (0.1 mg/kg), both given intravenously, and then positioned in lateral recumbency with the affected joint uppermost. A small window of 4x4cm area surrounding the greater trochanter was clipped and aseptically prepared. The limb was then placed parallel to the table surface and in a neutral position by an assistant, and the clinician (the same in all procedures) inserted a 21-gauge with 2.5″ length needle, just dorsal to the greater trochanter and perpendicular to the long axis of the limb [34]. Confirmation of correct needle placement was obtained through the collection of synovial fluid. All animals received 3 ml of platelet concentrate *per* hip joint, prepared with the commercially available V-PET kit (PALL Corporation), according to the manufacturer’s instructions. Fifty-five milliliters of whole blood were collected from the jugular vein of the patient, and then introduced into the provided closed system. The blood then flowed by action of gravity through the filter, where platelets where concentrated. The final product was collected using the provided syringe. After treatment, animals were rested for 3 consecutive days and resumed their normal activity over a period of 5 days. Signs of exacerbated pain, persistent stiffness of gait and changes in posture exhibited by the dogs, were evaluated by the veterinarian on the days 1 and 3 after the IA procedure. If no complaints were registered, the animal could resume its normal activity [35, 36].

Response to treatment, as measured by the CBPI (Additional file 1), COI (Additional file 2), LOAD (Additional file 3) and HVAS (Additional file 4) was evaluated before treatment (T0), after 15 days (T1) and 1 (T2), 2 (T3), 3 (T4), 4 (T5), 5 (T6) and 6 (T7) months after initial treatment. These were completed by the trainers, who were unaware of which treatment the animal received, and after receiving the published instructions for each for them. Normality was assessed with a Shapiro-Wilk test and each instant was compared with T0 with a Paired Samples T-Test. All results were analyzed with IBM SPSS Statistics version 20 and a significance level of *p* < 0.05 was set.

## Results

All animals enrolled were followed during a 6-month evaluation period. The sample included 15 working dogs (*N* = 15) of both genders (8 females and 7 males), with a mean age of 7 ± 2.4 years old and body weight of 31.1 ± 4.57 kg. Four breeds were represented: German Shepherd Dogs (*n* = 10), Labrador Retriever (*n* = 3), Belgian Malinois Shepherd Dogs (*n* = 1) and Catch Dog of São Miguel (*n* = 1). These were use of force, product and human scent dogs in active work at the time of treatment and during the follow up period, and where in similar kennels of the Portuguese Gendarmerie Canine Unit. Each animal received an average total solution volume of 6 ml of platelet was produced with V-PET, divided in 3 ml per hip joint. Increased lameness after the IA administration was observed in four dogs, which was spontaneously resolved within 48 h.

When comparing results between each time moment and T0, significant differences were observed in all moments and with different CMIs. With HVAS, significant improvements were observed at T1 (*p* < 0.01), T2 (*p* = 0.02) and T3 (*p* < 0.01). When considering individual results, improved results were observed in 12 animals at T1 (80%), 11 at T2 (73.3%), 14 at T3 (93.3%), 9 at T4-T6 (60%) and 8 at T7 (53.3%).

**Fig. 1:**
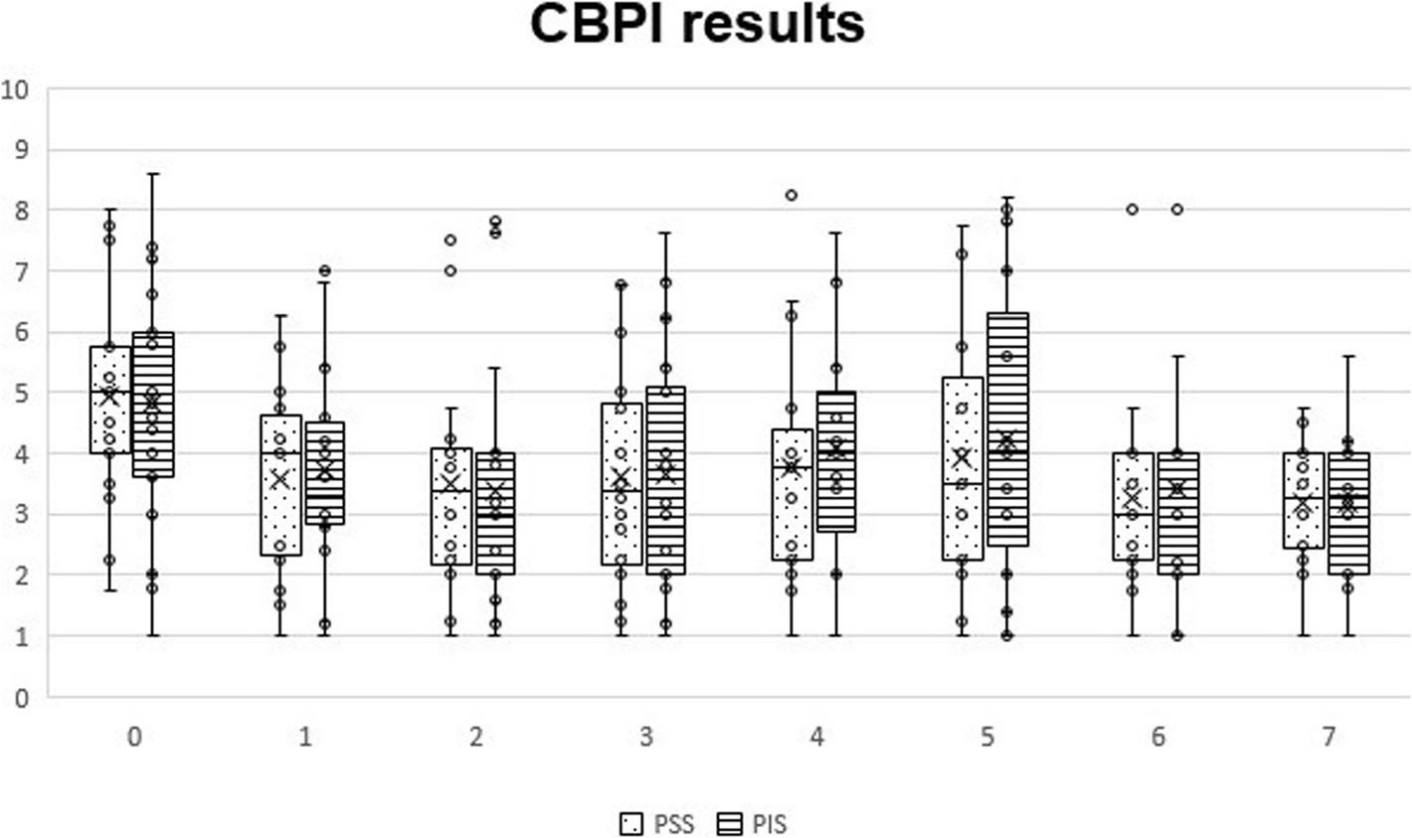
Overall Canine Brief Pain Inventory scores, by section and instant. Box plots represent median, 25th and 75th percentiles, and whiskers represent 10th and 90th percentiles

With CBPI, significant differences were observed at T1 (*p* < 0.01 for PSS and *p* = 0.01 for PIS), T2 (*p* < 0.01 for PSS and PIS), T3 (*p* < 0.01 for PSS and PIS), T4 (*p* < 0.01 for PSS and *p* = 0.03 for PIS), T5 (*p* = 0.03 for PSS and PIS), T6 (*p* < 0.01 for PSS and *p* = 0.04 for PIS) and T7 (*p* < 0.01 for PSS and *p* < 0.05 for PIS). Evolution of PSS and PIS scores can be observed in Fig. 1. Individual treatment success, as measured by the CBPI, has been defined as a reduction of ≥1 in PSS and ≥ 2 in PIS [37]. Treatment was successful in reducing PSS in 8 animals at T1 (53.3%), 11 at T2 (73.3%), 10 at T3 (66.7%), 9 at T4 (60%) and 8 at T5-T7 (53.4%). In addition, scores improved for 10 animals at T1 (66.7%), 12 at T2 (80%), 11 at T3 (73.3%), 10 at T4 (66.7%), 12 at T5 (80%) and 11 at T6-T7 (73.3%). Considering PIS, treatment was a success in 4 animals at T1 (26.7%), 5 at T2 (33.3%), 4 at T3 (26.7%), 3 at T4-T5 and T7 (20%,) and 4 at T6 (26.7%). Treatment also improved scores for 10 animals at T1 (66.7%), 11 at T2-T3 (73.3%), 9 at T4 (60%), 10 at T5 (66.7%) and 8 at T6-T7 (53.3%).

**Fig. 2:**
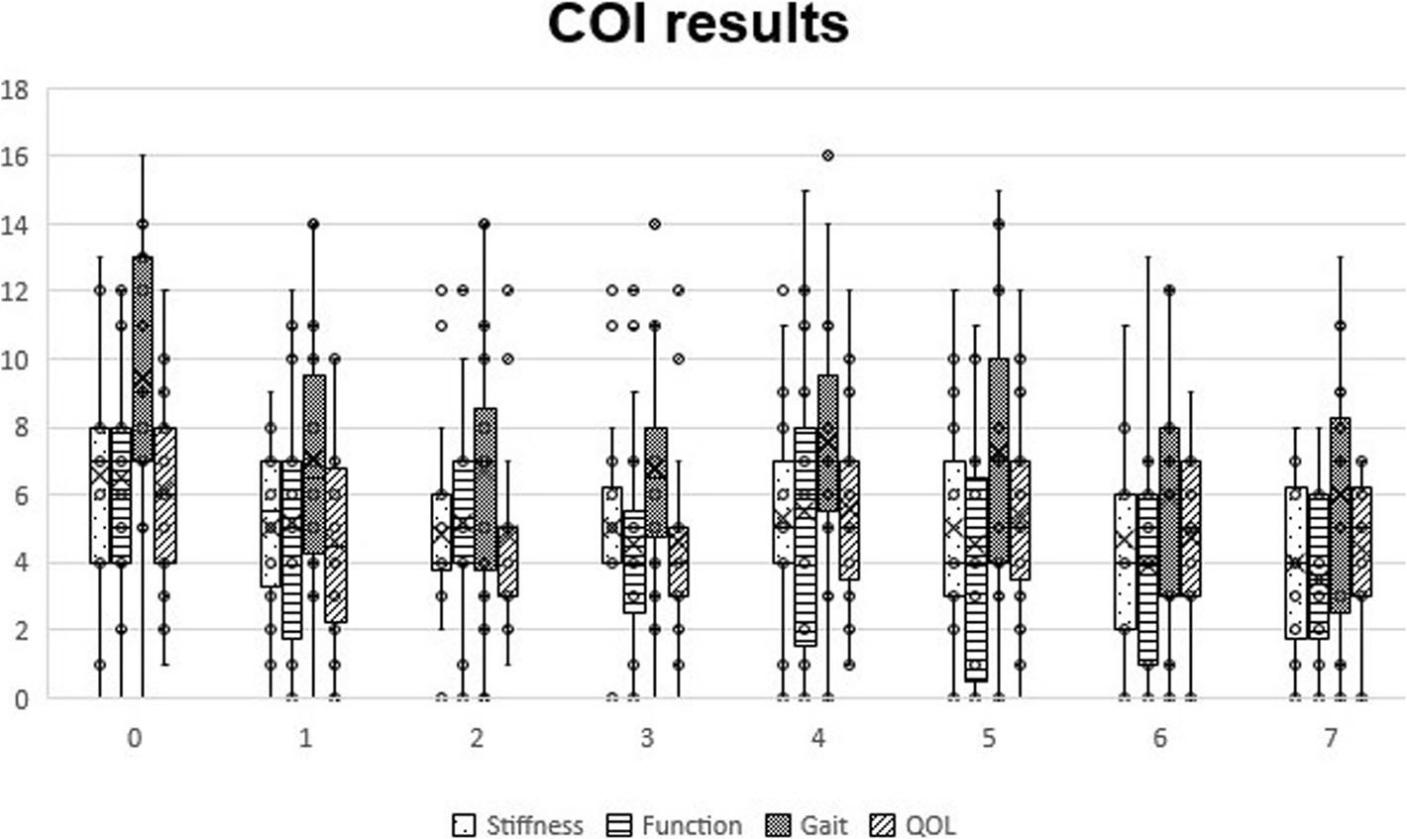
Canine Orthopedic Index scores, by domain and instant. Box plots represent median, 25th and 75th percentiles, and whiskers represent 10th and 90th percentiles

With COI, significant differences were observed at T1 (*p* < 0.01 for COI, Gait and QOL, *p* = 0.02 for Function and *p* < 0.05 for Stiffness), T2 (*p* < 0.01 for Gait, *p* = 0.01 for COI, *p* = 0.02 for Function and QOL, and *p* = 0.04 for Stiffness), T3 (*p* < 0.01 for Function and Gait, *p* = 0.01 for COI, and p = 0.02 for QOL), T4 (*p* = 0.03 for Gait), T5 (p < 0.01 for COI, Function and Gait, and *p* = 0.03 for Stiffness), T6 (*p* < 0.01 for Function and Gait and *p* < 0.05 for COI) and T7 (*p* < 0.01 for Function and Gait and *p* = 0.01 for COI). Evolution of COI scores can be observed in Fig. 2. When considering individual results, an improvement was observed in all dimensions in all evaluation points. Regarding stiffness, 7 animals recorded better scores at T1 (46.7%), 6 at T2-T4 (40%) and 7 at T5-T7 (46.7%). Function scores improved in 6 animals at T1-T2 (40%), 8 at T3-T4 (53.3%), 11 at T5 (73.3%), 9 at T6 (60%) and 8 at T7 (53.3%). Gait scores also improved in a large majority of animals, with better results when comparing to baseline being registered in 13 animals at T1 (86.7%) at T1, 11 at T2 (73.3%), 10 at T3 (66.7%), 9 at T4 (60%) and 10 at T5-T7 (66.7%). Regarding QOL, 11 animals recorded better scores at T1 (73.3%), 7 at T2 (46.7%), 9 at T3 (60%), 7 at T4-T5 (46.7%) and 6 at T6-T7 (40%). Several animals also showed better overall COI scores, namely 13 animals recorded better scores at T1 (86.7%), 10 at T2 (66.7%), 11 at T3 (73.3%), 10 at T4-T6 (66.7%) and 11 at T7 (73.3%).

No significant differences were registered in the LOAD scores when comparing each moment with T0. When considering individual results, an improvement in results was observed in 8 animals at T1 (53.3%), 10 at T2 (66.7%), 11 at T3 (73.3%), 12 at T4 (80%), 11 at T5 (73.3%) and 10 at T6-T7 (66.7%).

## Discussion

OA is a common, incurable condition that, despite extensive research, still has limited treatment options available [10, 38, 39]. Its management is largely palliative, focussing on the alleviation of symptoms, mainly pain, and slowing down the progression of the disease [40, 41]. The results show that the animals included in this sample experienced significant improvements for several months, as measured with several validated CMI. Since there is a similarity in neurophysiology paths across mammals, which indicates that pain is experienced by humans and animals in similar ways [42], it is reasonable that these results could also be observed in humans.

Previous reports in dogs have described that a single IA autologous platelet therapy injection has resulted in clinical improvements for 12 weeks, in some cases without progression of radiographic signs [27, 31]. Our results show that significant improvements, when compared to baseline values, are still present at the 6-month evaluation point, a considerably longer period.

The CBPI survey is often the test of choice to evaluate chronic pain in veterinary medicine [43, 44]. Treatment success in OA dogs has been set as a decrease in PSS ≥ 1 and in PIS ≥ 2 [37, 45]. Our results show that IA autologous platelet therapy was able to significantly reduce pain levels in dogs, in some cases up to 6 months. Interestingly, it was also able to significantly reduce pain interference scores, in contrast to other treatment modalities, such as NSAIDs and nutraceuticals [46].

LOAD was initially developed to assess dog with elbow OA, but was latter deemed as reliable to asses canine OA in general [16]. It has shown good reliability, just lower than peak vertical force generated by a force platform, although both results correlate. CBPI and LOAD results have a moderate correlation [15, 16]. Even though improvements in individual LOAD scores have been observed, no significant differences when considering the entire sample was considered. A possible explanation to this fact may be in the nature of the dogs included in the sample and of the CMI itself. Many of the LOAD items focus on the level of activity of the dog, its willingness or ability to exercise. Since the animals included in this study are working dogs with a very high work drive, it is possible that the constant willingness of these animals to exercise, even in cases of overt lameness and pain, may have led to smaller variations in LOAD scores, when compared to other CMIs. This may also be true for PIS scores, in addition to the fact that were low to begin with for many patients, making it harder to reach a significant reduction.

Considering COI results, it was also interesting to observe that significant improvements were observed up to the last evaluation point, specifically in overall score but also gait and function, two areas particularly affected by OA. Individual results in all dimensions improved for most animals, in many cases up to T7.

Visual analogue scales are one of the techniques used to score pain and assess its severity, allowing to compare different analgesic protocols. The Hudson Visual Analogue Scale (HVAS) has been deemed as repeatable and valid to assess the degree of mild to moderate lameness in dogs, compared with force plate analysis as a criterion-referenced standard [20]. In this study, significant variations in HVAS scores were observed, up to T3, even though individual results improved for a majority of animals during the 6-month evaluation period.

The obtained results give strength the concept that different components of OA are captured by different CMI [16], and reinforce the advantage of using more than one of them when monitoring patients and response to treatment. As a whole, CMIs represent a patient-centred approach, similar in human and veterinary medicine [10]. It is still unknown if respondents should be permitted or not to see previous answers. Previous reports show little difference has been observed between both approaches, but allowing responders to see previous answers results in increased treatment effect sizes, which may increase clinical trial power [47]. In this study, in order to reduce bias, trainers were not allowed to see previous answers, as it might influence their responses, particularly with a long follow-up period.

Increased lameness was observed in four dogs, which spontaneously resolved within 48 h. This is in contrast to what is observed with NSAIDs, often the first line of treatment but with well documented side-effects, particularly when for long periods [48]. It was, however, in line with what has been described in humans, were platelet concentrates can produced local and transient side-effects, such as injection pain and local inflammation, that take 2–10 days to resolve [26, 49, 50]. No additional medication was administered to the animals during the follow up period.

This study presents some limitations, namely the lack of a control group. Even though the validity of the results is reinforced by the use of several CMIs, further studies should include other evaluation method such as Force Plait Gait Analysis or Stance Analysis. Future studies should also evaluate the effect that both different cell composition and administration frequencies have on clinical results.

## Conclusions

Autologous platelet therapy showed to be a promising treatment option for the treatment of OA, as this naturally occurring canine model experienced significant improvements, up to the 6-month follow up moment. Further studies are required, particularly to determine the clinical effect of different administration frequencies.

### Competing interests

The V-PET kits used in this study were provided by the Pall Corporation.

## Supplementary information

**Additional file 1.** Appendix a – the canine brief pain inventory.

**Additional file 2.** Appendix b – the canine orthopedic index.

**Additional file 3.** Appendix c – liverpool osteoarthritis in dogs.

**Additional file 4.** Appendix d – hudson visual analogue scale.

## Data Availability

The datasets generated and/or analysed during the current study are not publicly available since all data generated or analysed during this study are included in this published article, but are available from the corresponding author on reasonable request.

## Abbreviations

CBPI: Canine Brief Pain Inventory
CMI: Clinical Metrology instruments
COI: Canine Orthopedic Index
HVAS: Hudson Visual Analogue Scale
LOAD: Liverpool Osteoarthritis in Dogs
OA: Osteoarthritis
PIS: Pain Interference Score
PSS: Pain Severity Score
QOL: Quality of Life
V-PET: Veterinary Platelet Enhancement Therapy
