# Supplementary material for: A report on the use of a single intra-articular administration of autologous platelet therapy in a naturally occurring canine osteoarthritis model - a preliminary study
Source: BMC Musculoskelet Disord. 2020 Feb 27;21:127. doi: 10.1186/s12891-020-3140-9 (PMC7047415; doi:10.1186/s12891-020-3140-9)
Supplement: Supplementary file 1 — Additional file 1. Appendix a – the canine brief pain inventory. [file 12891_2020_3140_MOESM1_ESM.pdf]

APPENDIX A – THE CANINE BRIEF PAIN INVENTORY

Description of Pain:

Rate your dog's pain. (0-10, 0 = no pain, 10 = extreme pain)

1. Which one number best describes the pain at its **worst** in the last 7 days.
2. Which one number best describes the pain at its **least** in the last 7 days.
3. Which one number best describes the pain at its **average** in the last 7 days.
4. Which one number best describes the pain as it is **right now**.

Description of Function:

Which one number best describes how during the past 7 days **pain has interfered** with your dog's (0-10, 0 = does not interfere, 10 = completely interferes):

5. General Activity
6. Enjoyment of Life
7. Ability to Rise to Standing From Lying Down
8. Ability to Walk
9. Ability to Run
10. Ability to Climb Up (for example Stairs or Curbs)

Overall Impression:

11. Which response best describes your dog's overall quality of life over **the last 7 days**?

(Bad, Fair, Good, Very Good Excelent)
