# Supplementary material for: A report on the use of a single intra-articular administration of autologous platelet therapy in a naturally occurring canine osteoarthritis model - a preliminary study
Source: BMC Musculoskelet Disord. 2020 Feb 27;21:127. doi: 10.1186/s12891-020-3140-9 (PMC7047415; doi:10.1186/s12891-020-3140-9)
Supplement: Supplementary file 2 — Additional file 2. Appendix b – the canine orthopedic index. [file 12891_2020_3140_MOESM2_ESM.pdf]

APPENDIX B – THE CANINE ORTHOPEDIC INDEX

Description of Stiffness:

The following questions concern the amount of joint stiffness your dog has experienced in the **past 7 days**.

Stiffness is the restriction or slowness in the ease with which your dog moves his/her joints.

Please select **one** answer for each question below.

1. How severe is your dog's stiffness after first wakening **in the morning**?
   - None;
   - Mild;
   - Moderate;
   - Severe;
   - Extreme.
2. **Later in the day**, how severe is your dog's stiffness after lying down for at least 15 minutes?
   - None;
   - Mild;
   - Moderate;
   - Severe;
   - Extreme.
3. How much of a problem does your dog have **rising to standing** after lying down for at least 15 minutes?
   - No problems;
   - Mild problems;
   - Moderate problems;

- Severe problems;
- Extreme problems.

4. In general, over the past 7 days, how much difficulty has your dog had with his or her joints?
   - None;
   - Mild;
   - Moderate;
   - Severe;
   - Extreme.

Description of Function:

Please indicate how much of a problem each of the following activities has been for your dog over the **past 7 days**.

Please select **one** answer for each question below.

5. **Jumping up** (as in getting into the car or onto the bed)?
   - No problems;
   - Mild problems;
   - Moderate problems;
   - Severe problems;
   - Extreme problems.

6. **Jumping down** (as in getting out of the car or off of the bed)?
   - No problems;
   - Mild problems;
   - Moderate problems;

- Severe problems;
- Extreme problems.

7. **Climbing up** (as in stairs, ramps or curbs)?
    - No problems;
    - Mild problems;
    - Moderate problems;
    - Severe problems;
    - Extreme problems.
8. **Climbing down** (as in stairs, ramps or curbs)?
    - No problems;
    - Mild problems;
    - Moderate problems;
    - Severe problems;
    - Extreme problems.

Description of Gait:

The following questions concern your dog's gait over **past 7 days**.

Gait refrs to the manner in which your dog uses its legs as it moves

Please select **one** answer for each question below.

9. On average, how severe was your dog's limp **during** mild activities (such as short walks)?
    - None;
    - Mild;
    - Moderate;
    - Severe;
    - Extreme.

10. On average, how severe was your dog's limp **<u>during</u>** mild activities (such as long walks, playing or running)?

- None;
- Mild;
- Moderate;
- Severe;
- Extreme.

11. How often did your dog limp **<u>the day after</u>** moderate activities (such as long walks, playing or running)?

- Never;
- Rarely;
- Occasionally;
- Frequently;
- Constantly.

12. How often have you been aware of your dog's joint problems?

- Never;
- Rarely;
- Occasionally;
- Frequently;
- Constantly.

13. How often did your dog dog 'pay' for over-activity, with increased pain or stiffness the following day?

- Never;
- Rarely;
- Occasionally;

- Frequently;
- Constantly.

Description of Quality of Life:

Please select **<u>one</u>** answer for each question below.

14. In the past 7 days, what has been your level of concern that your dog's joint problems will shorten his or her life?
    - None;
    - Mild;
    - Moderate;
    - Severe;
    - Extreme.
15. In the past 7 days, what has been your level of concern that your dog is generally slowing down?
    - None;
    - Mild;
    - Moderate;
    - Severe;
    - Extreme.
16. Overall, how would you rate your dog's quality of life over the past 7 day?
    - Excellent;
    - Very Good;
    - Good;
    - Fair;
    - Poor.
