# Supplementary material for: A report on the use of a single intra-articular administration of autologous platelet therapy in a naturally occurring canine osteoarthritis model - a preliminary study
Source: BMC Musculoskelet Disord. 2020 Feb 27;21:127. doi: 10.1186/s12891-020-3140-9 (PMC7047415; doi:10.1186/s12891-020-3140-9)
Supplement: Supplementary file 3 — Additional file 3. Appendix c – liverpool osteoarthritis in dogs. [file 12891_2020_3140_MOESM3_ESM.pdf]

APPENDIX C – LIVERPOOL OSTEOARTHRITIS IN DOGS

Mobility:

Generally

1. How is your dog's mobility in general?

   - Very good;

   - Good;

   - Fair;

   - Poor;

   - Very poor.

2. How disabled is your dog by his/her lameness?

   - Not at all disabled;
   - Slightly disabled;
   - Moderately disabled;
   - Severely disabled;
   - Extremely disabled;

3. How active is your dog?

   - Extremely active;
   - Very active;
   - Moderately active;
   - Slightly active;
   - Not at all active;

4. What is the effect of cold, damp weather on your dog's lameness?

   - No effect;
   - Mild effect;
   - Moderate effect;
   - Severe effect;
   - Extreme effect;

5. To what degree does your dog show stiffness in the affected leg after a 'lie down'?

   - No stiffness;
   - Mild stiffness;
   - Moderate stiffness;
   - Severe stiffness;
   - Extreme stiffness;

At exercise

6. At exercise, how active is your dog?

   - Extremely active;
   - Very active;
   - Fairly active;
   - Not very active;
   - Not at all active;

7. How keen to exercise is your dog?

   - Extremely keen;
   - Very keen;

- Fairly keen;
- Not very keen;
- Not at all keen.

8. How would you rate your dog's ability to exercise?
   - Very good;
   - Good;
   - Fair;
   - Poor;
   - Very poor;

9. What overall effect does exercise have on your dog's lameness?
   - No effect;
   - Mild effect;
   - Moderate effect;
   - Severe effect;
   - Extreme effect.

10. How often does your dog rest (stop/sit down) during exercise?
    - Never;
    - Hardly ever;
    - Occasionally;
    - Frequently;
    - Very frequently.

11. What is the effect of cold, damp weather on your pet's ability to exercise?
   - No effect;
   - Mild effect;
   - Moderate effect;
   - Severe effect;
   - Extreme effect.

12. To what degree does your dog show stiffness in the affected leg after a 'lie down' following exercise?
   - No stiffness;
   - Mild stiffness;
   - Moderate stiffness;
   - Severe stiffness;
   - Extreme stiffness;

13. What is the effect of your dog's lameness on his/her ability to exercise?
   - No effect;
   - Mild effect;
   - Moderate effect;
   - Severe effect;
   - Extreme effect.
